# Kamerabasierte Navigation im Hybridoperationssaal

**DOI:** 10.1007/s00104-022-01777-7

**Published:** 2022-12-09

**Authors:** Mathis Wegner, Andreas Seekamp, Sebastian Lippross

**Affiliations:** Orthopädie und Unfallchirurgie, Universitätsklinikum Schleswig-Holstein, Campus Kiel, Arnold-Heller-Str. 3, 24105 Kiel, Deutschland

**Keywords:** Wirbelsäulenchirurgie, Cone-beam-Computertomographie, Augmented reality surgical navigation, Volumentomographie, Oberflächenreferenzierung, Spine surgery, Cone beam computed tomography, Augmented reality surgical navigation, Volume tomography, Surface referencing

## Abstract

Kamerabasierte Navigation im Hybridoperationssaal stellt in der Wirbelsäulenchirurgie eine Möglichkeit der präzisen, komplikationsarmen und effizienten Implantation von Osteosynthesematerial dar. Neben der Erhöhung der Patientensicherheit verringert sich bei Nutzung einer kamerabasierten Navigation als Orientierungshilfe für den Chirurgen die Strahlenbelastung. Im Mittelpunkt der kamerabasierten Navigation stehen die anatomischen Landmark-Kenntnisse des Chirurgen, die präoperative Bildakquise und die folgende Informationsintegration durch die eingesetzte Planungssoftware. Die gelieferten Informationen aus Volumentomographie (Cone-beam-Computertomographie, CBCT) und Oberflächenreferenzierung durch den Video-Input von vier optischen Kameras und den dazugehörigen Oberflächenmarkern werden durch den Einsatz einer Software gesammelt, prozessiert, optimiert und individuell angepasst. Das Ergebnis ist die Erstellung einer Trajektorie, welche dem Operateur die leichtere Analyse und Evaluation komplexer anatomischer Strukturen und die erleichterte Durchführung des geplanten Eingriffs ermöglichen. Die minimal-invasive Insertion von Pedikelschrauben mithilfe einer oberflächenreferenzierten Navigation („augmented reality surgical navigation“, ARSN) bietet eine vergleichbare Genauigkeit zur konventionellen fluoroskopischen Insertion von Pedikelschrauben bei gleichzeitiger Strahlungsreduktion durch den Verzicht auf eine postoperative computertomographische Bildgebung.

## Abstract (en)

Camera-based navigation in the hybrid operating room represents a possibility for precise, low-complication and efficient pedicle screw insertion in spinal surgery. In addition to increasing patient safety, the use of camera-based navigation as an orientation aid for the surgeon reduces radiation exposure. Camera-based navigation focuses on the surgeon’s anatomical knowledge of landmarks, preoperative image acquisition, and subsequent information integration by the navigation software. The information provided from volume tomography (cone beam computed tomography, CBCT) and surface referencing through the video input from four optical cameras and the associated surface markers is collected, processed, optimized and customized by the software used. The result is the creation of a trajectory that allows the surgeon to analyze and evaluate complex anatomical structures more easily and facilitates the performance of the planned procedure. Minimally invasive insertion of pedicle screws using surface-reference navigation (augmented reality surgical navigation; ARSN) provides comparable accuracy to conventional fluoroscopic insertion of pedicle screws while reducing radiation by eliminating the need for postoperative computed tomographic imaging.

## Hybridoperationssaal

Hochmoderne Hybridoperationssäle stehen für eine neue Dimension der interdisziplinären Zusammenarbeit in chirurgischen Fächern. Am Universitätsklinikum Schleswig-Holstein (UKSH), Campus Kiel wird der Hybridoperationssaal nach initialer Installation 2014 für den Einsatz in der Herz- und Gefäßchirurgie bei orthopädischen und unfallchirurgischen Operationen seit 2015 regelmäßig eingesetzt. Braun et al. zeigten 2019, dass in der Wirbelsäulen- und Beckenchirurgie bei gleichen Operationszeiten eine signifikante Reduktion der Revisionseingriffe erreicht werden konnte [1].

**Figure 1:**
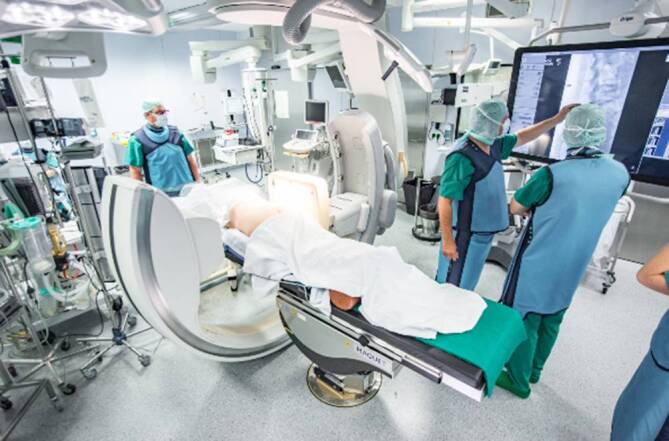

Die Besonderheit des verwendeten Systems am UKSH, Campus Kiel (Allura Xper FD20 mit FlexMove; Fa. Philips, Best, Niederlande) besteht in der Montage des isozentrischen C‑Bogens an der Decke des Hybridoperationssaals bei fest montiertem Operationstisch, was eine intuitive und flexiblere Steuerung der Röntgeneinheit durch ein Deckenschienensystem erlaubt. Die Steuerung kann ohne Probleme steril an einer mobilen Steuerungseinheit durch den Operateur erfolgen. Die 3‑D-Rekonstruktion im Hybridoperationssaal erfolgt im Multi-Provider-Router(MPR)-Format bei geringer Scanzeit aufgrund der Deckenmontage (Abb. 1).

## Cone-beam-Computertomographie

Durch die Cone-beam-Computertomographie (CBCT) kann eine Navigation sowohl mittels 2‑D-Fluoroskopie als auch 3‑D-Navigation erzeugt werden. Durch die Rotation eines konusförmigen Strahlenbündels um das abzubildende Objekt wird eine Volumentomographie erzeugt, welche als multiplanare Rekonstruktion oder als Volumengrafik dargestellt wird. Die Erstellung des 3‑D-Datensatzes erfolgt aus 230 Röntgenprojektionen, welche während einer ca. 10 s dauernden 180°-Rotation um das abzubildende Objekt generiert werden. Die abgebildete Region entspricht den Ausmaßen 25 × 25 cm in der axialen Ebene bei einer kraniokaudalen Ausdehnung von 19,5 cm [2]. Dieser dreidimensionale Datensatz kann zur Navigation bei wirbelsäulenchirurgischen Eingriffen genutzt werden. Gegenüber einer konventionellen CT-Bildgebung bestehen stärkere durch Streustrahlung bedingte Bildartefakte [3].

**Figure 2:**
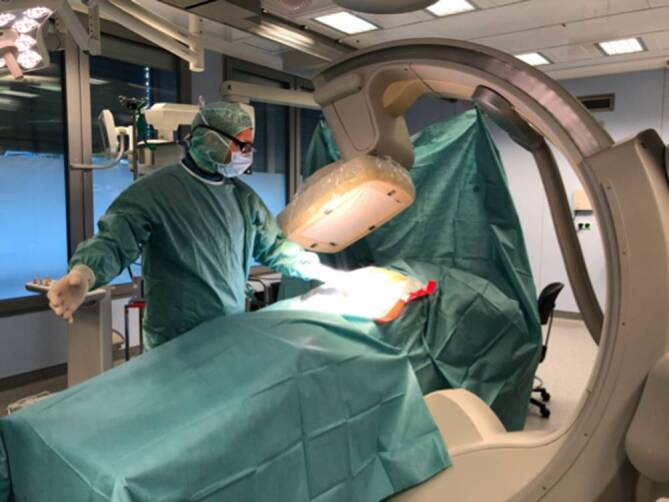

Am UKSH, Campus Kiel findet die CBCT regelmäßigen Einsatz und wird zur Planung und Navigation in der Wirbelsäulenchirurgie angewandt (Brainlab AG, München, Deutschland). Für die unfallchirurgischen Eingriffe am Becken und an der Wirbelsäule wird ein 3‑D-Protokoll genutzt, bei dem ein sog. Roll-Scan verwendet wird. Dabei wird der C‑Bogen seitlich um den Operationstisch geführt und die Anatomie in das Isozentrum des C‑Bogens positioniert. Die 230 Einzelaufnahmen über einen Winkelbereich von ca. 190° werden an einem 3‑D-Rechner zu einem Volumendatensatz rekonstruiert (Abb. 2).

## „Augmented reality surgical navigation“

Die Erzeugung von Trajektoriesystemen durch kamerabasierte Navigation im Hybridoperationssaal ist eine Möglichkeit der intraoperativen Navigierungshilfe. Im Set-up des Hybridoperationssaals mit einer CBCT zur Planung unterstützen oberflächenreferenzierte Navigationssysteme („augmented reality surgical navigation“ mit Tip-Tracking-Funktion, ARSN) die Implantation von Osteosynthesematerialien. Das ARSN-System basiert auf dem Video-Input von vier optischen Kameras, die in den Rahmen des C‑Bogen-Detektors eingebaut sind. Es erkennt Marker auf der Körperoberfläche des Patienten und den genutzten Operationsinstrumenten, kann diese Operationsinstrumente erfassen und deren Richtung und Eindringtiefe auf den 3‑D-Datensatz übertragen. Resultat ist die Erzeugung einer Virtual Reality, welche auf einem Bildschirm angezeigt wird. Voraussetzung für die Trajektorieerzeugung ist die kontinuierliche Videoerfassung flacher, kreisförmiger Klebemarker auf der Oberfläche des Patienten am Rande des Operationsfeldes und den kalibrierten Operationsinstrumenten. Diese sollten asymmetrisch angebracht werden, um die Referenzierung und Navigation nicht zu beeinträchtigen.

**Figure 3:**
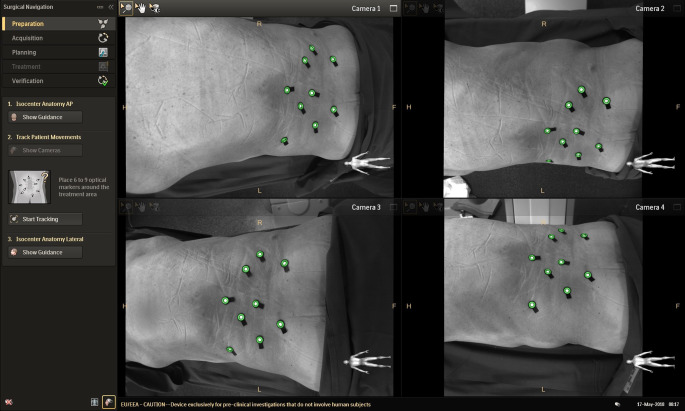

Anhand der erzeugten CBCT-Datensätze kann nach Festlegung anatomischer Strukturen durch den Operateur der Weg der einzubringenden Schrauben geplant werden. Der Insertionspunkt, die Position in der sagittalen, frontalen und axialen Ebene, die Schraubenlänge und der Schraubendurchmesser können aufgrund der Planung festgelegt werden und während der Operation durch ARSN bei Insertion überprüft werden. Bei dem am UKSH, Campus Kiel verwendeten System handelt sich um die von Philips Medical Systems entwickelte „Surgical Navigation R1.5“ (Philips Medical Systems DMC GmbH, Hamburg, Deutschland) Softwareanwendung (Abb. 3 und 4).

**Figure 4:**
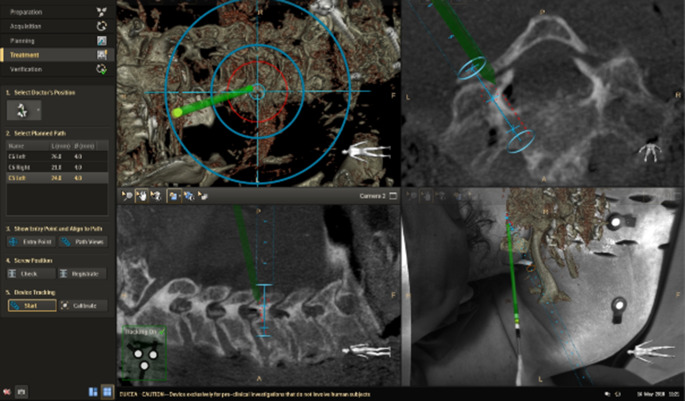

## Diskussion

Durch die Integration eines Flachbild-C-Bogens und der Verwendung von Volumentomographien (CBCT) im Operationssaal [2] kann im Hybridoperationssaal eine genauere intraoperative Darstellung der Wirbelsäule vor und nach Insertion von Pedikelschrauben erfolgen. Durch die Möglichkeit der dreidimensionalen Darstellung der Wirbelsäule sinkt die Zahl der Revisionsoperationen, da mögliche Implantatfehllagen intraoperativ detektiert werden können [1]. Als fester Bestandteil bei komplizierten unfallchirurgischen Operationen findet durch die Durchführung unfallchirurgischer Operationen im Hybridoperationssaal [4] unter Verwendung von in der CBCT erzeugten dreidimensionalen Bildsätzen eine signifikante Strahlungsreduktion durch den möglichen Verzicht auf eine postoperative Computertomographie statt [5].

Die CBCT ermöglicht eine gute Schraubenpositionierung bei signifikanter Strahlungsreduktion

Speziell bezogen auf die Implantation von Pedikelschrauben bei Wirbelsäulenoperationen konnte durch die Verwendung einer CBCT zur präoperativen Planung eine sehr gute Schraubenpositionierung unter signifikanter Strahlungsreduktion nachgewiesen werden. Insgesamt wurden in der Studie von Peh et al. 2022 [6] sieben Kadaver an der thorakalen und lumbalen Wirbelsäule mit Pedikelschrauben instrumentiert [7]. Zweihunderteinundvierzig kanülierte Pedikelschrauben wurden perkutan eingebracht: 3 (1 %), 168 (70 %) und 70 (29 %) in der Hals‑, Brust- bzw. Lendenwirbelsäule. Es wurde intraoperativ eine fluoroskopische CBCT-Bildgebung durchgeführt und im weiteren Verlauf als Referenz eine konventionelle CT-Bildgebung. Anhand des Gertzbein-Robbins-Gradings (GRS; [8]) wurde die Schraubenlage durch drei unabhängige Radiologen evaluiert. Zwischen CBCT und CT wurde eine nahezu perfekte Übereinstimmung bei der Beurteilung der Pedikelschraubenlage festgestellt. Somit stellt eine Bildgebung mittels intraoperativer CBCT mit einem C‑Bogen in einem Hybridoperationssaal eine sehr zuverlässige Identifizierungsmethode der Schraubenplatzierung da und kann die Strahlungsdosis durch den Verzicht auf eine konventionelle postoperative CT-Bildgebung erheblich reduzieren.

Elmi-Terradner et al. konnten unter Verwendung von ARSN bei 253 thorakalen und lumbosakralen Pedikelschrauben bei 20 Patienten eine Genauigkeit der Pedikelschraubenimplantation von 94,1 % erzeugen. Gemessen wurde die Genauigkeit ebenfalls anhand des GRS. Es wurde keine Schraubenfehllage identifiziert, wobei eine akzeptable Insertionszeit erreicht wurde [9]. Peh et al. konnten in einer Kadaverstudie die konventionelle fluoroskopische Technik zur perkutanen Insertion von Pedikelschrauben mit einer oberflächenreferenzierten Navigation (ARSN mit Tip-Tracking-Funktion) vergleichen [7]. Ausgewertet wurden hierbei die Durchführbarkeit der ARSN im Hybridoperationssaal, ob ein Unterschied in der Genauigkeit der Schraubenlage im Pedikel zwischen den beiden Methoden bestand und ob sich die beiden Methoden in der Operationszeit und der verwendeten Strahlung unterscheiden. Alle Operationen wurden von einem einzigen erfahrenen Operateur durchgeführt. Die Genauigkeit wurde anhand einer postoperativen Computertomographie durch drei Rater mittels GRS festgelegt. Signifikante Unterschiede konnten in den angelegten Parametern in dieser Studie nicht gezeigt werden. Inkludiert man die Vorbereitungszeit der ARSN verlängert sich die Gesamtzeit der Operation bei vergleichbarer Schraubenlage. Ein Vorteil bezüglich der Genauigkeit der Schraubenlage konnte insgesamt nicht gezeigt werden [10, 11]. Die Reduktion der Strahlenbelastung stellt einen Vorteil des Verfahrens dar.

## Fazit für die Praxis

- „Augmented reality surgical navigation“ (ARSN) im Hybridsaal ist eine wenig invasive Operationsmethode, die sich gut in den klinischen Alltag implementieren lässt.
- Aktuelle Untersuchungen zeigen, dass die Genauigkeit der Schraubenplatzierung an der Wirbelsäule mindestens vergleichbar mit konventionell-röntgengestützten Methoden ist.
- Die Operation ist strahlungsarm durchführbar.
- Die prä- und postoperative Cone-beam-Computertomographie (CBCT) ist für den Patienten unvermeidbar, allerdings erfolgt eine intraoperative Lagekontrolle, die eine weitere CT-Bildgebung unnötig macht.
